# The Influence of Adalimumab and Cyclosporine A on the Expression Profile of the Genes Related to TGF*β* Signaling Pathways in Keratinocyte Cells Treated with Lipopolysaccharide A

**DOI:** 10.1155/2020/3821279

**Published:** 2020-07-26

**Authors:** Iwona Adwent, Beniamin Oskar Grabarek, Marta Kojs-Mrożkiewicz, Ryszard Brus, Rafał Staszkiewicz, Andrzej Plewka, Michał Stasiowski, Anita Lyssek-Boroń

**Affiliations:** ^1^Department of Histology, Cytophysiology and Embryology, Faculty of Medicine in Zabrze, University of Technology in Katowice, Poland; ^2^Department of Dermatology, Andrzej Mielecki Memorial Independent Public Clinical Hospital, Medical University of Silesia, Katowice, Poland; ^3^5th Military Clinical Hospital with the SP ZOZ Polyclinic, Krakow, Poland; ^4^Department of Nurse, High School of Strategic Planning, Koscielna 6, 41-303 Dąbrowa Górnicza, Poland; ^5^Institute of Health Sciences, University of Opole, Poland; ^6^Department of Anaesthesiology and Intensive Therapy, SMDZ in Zabrze, Medical University of Silesia, Katowice, Poland; ^7^Department of Ophthalmology with Paediatric Unit, St. Barbara Hospital, Trauma Center, Sosnowiec, Poland; ^8^Department of Ophtamology, Faculty of Medicine in Zabrze, University of Technology in Katowice, Poland

## Abstract

**Background:**

In the treatment of moderate to severe psoriasis, cyclosporine A (CsA) conventional therapy is used and biological, anti-cytokine treatment using, for example, anti-TNF drug—adalimumab.

**Aim:**

This study aimed at investigating the effect of CsA and adalimumab on the profile of mRNAs and protein expression associated with transforming growth factor *β* (TGF*β*) pathways in human keratinocyte (HaCaT) culture previously exposed to lipopolysaccharide (LPS).

**Materials and Methods:**

HaCaT culture was exposed to 1 ng/ml LPS for 8 hours+8 *μ*g/ml adalimumab for 2, 8, and 24 hours or 1 ng/ml LPS for 8 hours+100 ng/ml CsA for 2, 8, and 24 hours and compared to the control culture. Sulphorodamine B cytotoxicity assay was performed. The expression profile of mRNA related to TGF*β* paths was indicated by microarray and RTqPCR analyses. The ELISA test was used to analyze changes on the proteome level. Statistical analysis consisted of ANOVA analysis and the post hoc Tukey test (*p* < 0.05).

**Results:**

The cytotoxicity test showed that LPS, adalimumab, and cyclosporine in the concentration used in this experiment did not have any cytotoxicity effect on HaCaT cells. The largest fold changes (FC) in expression in (∣FC | >4.00) was determined for *TGFβ1-3*, *TGFβRI-III*, *SKIL*, *SMURF2*, *SMAD3*, *BMP2*, *BMP6*, *JAK2*, *UBE2D1*, *SKP2*, *EDN1*, and *PRKAR2B* (*p* < 0.05). In addition, on the protein level, the direct changes observed at mRNA were the same.

**Conclusion:**

Analysis of the microarray expression profile of genes associated with TGF*β* signaling pathways has demonstrated the potential of cyclosporin A and adalimumab to induce changes in their transcriptional activity. The anti-TNF drug seems to affect TGF*β* cascades to a greater extent than cyclosporin A. The obtained results suggest that the regularity of taking the drug is important for the efficacy of psoriasis therapy.

## 1. Introduction

The family of transforming growth factor beta (TGF*β*) is involved in the regulation of key cellular processes [1, 2]. The influence of TGF*β* has been described and confirmed in the regulation of cell proliferation as well as the cell cycle [3, 4], cell differentiation [5], and programmed cell death [6–9].

There are 5 TGF*β* isoforms; however, only the first three isoforms occur in humans [7, 10]. The biological effects exerted by this cytokine are the result of its interaction with TGF*β*RI-III receptors. TGF*β*1 is characterized by the highest expression in human tissues among all cytokine isoforms. It plays an important role in the induction and pathogenesis of psoriasis, during which its elevated level is observed [11, 12]. Depending on the biological context, including changes in the level of secretion of interleukins (IL), IL-1, IL-6, and IL-8, TGF*β* may inhibit or promote the proliferation of keratinocytes [13, 14]. Despite observations [15, 16], including ours [17, 18], that TGF*β* may become a complementary molecular marker in the diagnosis of the severity of the psoriatic process [15–18], the exact mechanism of its action is still not fully understood.

It is worth noticing that according to the gene ontology, genes related to the TGF*β* family are involved in the regulation of 52 different signaling pathways according to the PANTHER 14.1 program based on the gene ontology and the overrepresentation test [19]. The relationship of TGF*β* is underlined, including the following signaling cascades: PI3K/Akt, Wnt, Hedgehog, Notch, tumor necrosis factor alpha (TNF-*α*), JAK/STAT, interferon gamma (IFN-*γ*), SMAD, and nuclear factor kappa beta (NF*κ*B) [20–23].

According to the recommendations of the Polish Dermatological Society dated 2018, cyclosporin A (CsA) and biological drugs, among others TNF-*α* inhibitors (adalimumab), are recommended for the treatment of moderate and severe cases of psoriasis [24, 25].

The molecular mechanism of action of CsA is associated with a decrease of IL-2 secretion. This is possible by immunostimulation of T lymphocytes, which is mediated by the cyclosporine-cyclophilin complex, which blocks the activity of calmodulin (CALN) [26–28]. TGF*β* also inhibits IL-2 secretion via the SMAD-dependent pathway (TGF*β*/SMAD3). Therefore, it seems important to identify changes in the expression of genes related to TGF*β* under the influence of CsA [29–31]. In turn, adalimumab binds to the soluble and membrane-bound form of TNF-*α* and inhibits the interaction of this cytokine with the TNFR1 and TNFR2 receptors, consequently inhibiting the inflammatory response [32]. Beck indicates that between TNF-*α* and TGF-*β*1, there is a statistically significant positive correlation [33]. It is also indicated that TGF*β* contributes to the increase in TNF-*α* expression; however, under the influence of CsA, the expression of TNF-*α* was suppressed [34]. However, under the influence of adalimumab therapy, the induction of the TGF*β* pathway and the severity of its secretion by macrophages have been observed. This confirms the pleiotropic nature of cytokine activity and that the success of therapy depends on many factors [35].

The aim of this study was to evaluate the influence of adalimumab and cyclosporine A on the expression profile of genes associated with TGF*β* signaling pathways in human keratinocyte cell line (HaCaT) after inducting an inflammatory process by lipopolysaccharide (LPS).

## 2. Material and Methods

### 2.1. Cell Culture and Sulphorodamine B Cytotoxicity Assay

Human keratinocyte cells (HaCaT; CLS Cell Lines Service, Eppelheim, Germany) were cultured in Dulbecco's modified Eagle's medium (DMEM), high glucose–4500 mg/L (Sigma-Aldrich, St. Louis, MO, USA) supplemented with 10% fetal bovine serum (FBS), penicillin (100 U/ml), streptomycin (100 mg/ml), and glutamine (2 mM) (Sigma-Aldrich, St. Louis, MO, USA) and maintained in a humidified atmosphere at 37°C with 5% CO_2_.

In the first stage of our research, the sulphorodamine B cytotoxity assay (Sigma-Aldrich, St. Louis, MO, USA, catalog number 3520-42-1) was performed in accordance with the protocol provided by the manufacturer. The absorption was measured at a wavelength of 490-530 nm. The absorbance results observed from a control culture were described as 100%, and the percentage of viable cells in the culture exposed to any agents was revealed compared the control culture of untreated by the LPS/drug human keratinocyte cells.

The cytotoxity assay was made for cell culture treated with lipopolysaccharide (LPS; Sigma Aldrich, Poznań, Poland) in three different concentrations: 1 *μ*g/ml, 2 *μ*g/ml, and 10 *μ*g/ml for, cells exposed to adalimumab (0.8 *μ*g/ml, 8 *μ*g/ml, and 80 *μ*g/ml), cyclosporine A (1 ng/ml, 10 ng/ml, and 100 ng/ml) in comparison with the untreated cells (control of the experiment).

In next step, based on our previous observation, human keratinocyte cells were exposed to LPS in concentration 1 *μ*g/ml for 8 hours [36] and next, 8 *μ*g/ml adalimumab and 100 ng/ml cyclosporine A was added (concentration corresponding to the average therapeutic concentration in blood plasma in patients with psoriasis) for 2, 8, and 24 hours. Untreated cells were a control culture [37, 38].

### 2.2. RNA Isolation

The first step of molecular analysis was isolation of complete RNA from cell cultures using the TRIzol reagent (Invitrogen Life Technologies, Carlsbad, CA, USA, catalog number 15596026) according to the manufacturer's recommendation. Extracts of ribonucleic acid were evaluated qualitatively and quantitatively and, next, classified to the molecular analysis.

### 2.3. Microarray Profile of Genes Expression

The second step was associated with evaluation of the expression pattern of mRNAs related to TGF*β* paths using expression microarray HG-U133A 2.0 (Affymetrix, Santa Clara, CA) and GeneChip™ 3′ IVT PLUS Reagent Kit and GeneChip™ HT 3′ IVTPLUS Reagent Kit (Thermo Fisher, catalog number 902416) according to the manufacturer's protocol.

The names of probes and number of their ID were found from the Affymetrix NetAffx™ Analysis Center database after entering the phrase “TGF beta signaling pathways” (http://www.affymetrix.com/analysis/index.affx; accessed on 10^th^ March 2020).

The microarray analysis consisted of synthesizing cDNA from RNA extracts, adding poly-A control, synthesizing of biotinyl aRNA and hybridization aRNA of specimens with microarray probes and data analysis. The detailed protocol for molecular analysis was also described in our previous studies [37, 38].

### 2.4. Quantitative Reverse-Transcription Polymerase Chain Reaction

In the second stage of molecular analysis, the RTqPCR reaction was carried out to confirm the changes in the transcriptional activity of the genes for which the differences in expression compared with the control (FC, fold change, the 2^-*∆∆*Ct^ method) obtained from microarray data were ∣FC | >4.00 and corresponding with *TGFβ1-3*, *TGFβRI-III*, *SKIL*, *SMURF2*, *SMAD3*, *BMP2*, *BMP6*, *JAK2*, *UBE2D1*, *SKP2*, *EDN1*, and *PRKAR2B.* Endogenous control was *β*-actin (*ACTB*).  Table 1 presents nucleotide sequences of primers used in RTqPCR. The thermal condition of RTqPCR was as follows: reverse transcription (45°C for 10 min), activation of the polymerase (95°C for 2 min), and 40 cycles including denaturation (95°C for 5 s), annealing (60°C for 10 s), and elongation (72°C for 5 s).

This was conducted with the use of the SensiFAST™ SYBR No-ROX One-Step Kit, (Bioline, London, UK) according to the protocol.

### 2.5. ELISA Assay

The next stage of the molecular analysis was associated with determining changes in the level of selected proteins related to TGF*β* paths. Enzyme-linked immunosorbent assay (ELISA) reaction was carried out in this part of the study as recommended in the protocol. All ELISA kits were purchased from Thermo Fisher Scientific Company (Table 2).

### 2.6. Statistical Analysis

The statistical analysis of the obtained microarray data was done using the Transcriptome Analysis Console (Thermo Fisher, USA) and STATISTICA 13 PL (Cracow, Poland) software in order to analyze the results we obtained from the RTqPCR, ELISA reaction. The first stage of statistical analysis was to determine the normality of the data distribution. For this purpose, the Shapiro-Wilk test was performed; the result of which (*p* > 0.05) indicated that further analysis should be carried out using parametric methods. ANOVA variance analysis was performed, followed by Tukey's post-hoc test when expression changes were statistically significant (*p* < 0.05).

## 3. Results

**Figure 1:**
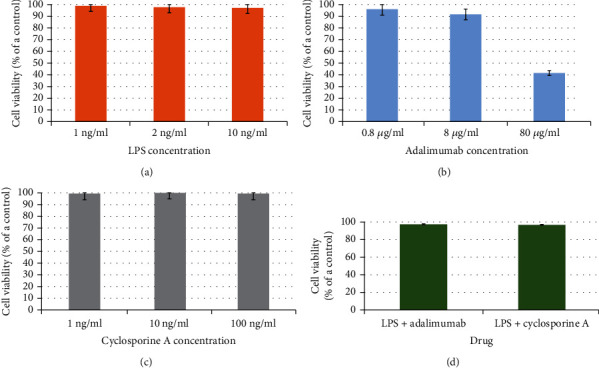
Results of cytotoxicity assay ((a) HaCaT with LPS, (b) HaCaT with adalimumab, (c) HaCaT with cyclosporine A, and (d) HaCaT with LPS and adalimumab or cyclosporine A).

The performed sulphorodamine B cytotoxic test did not showed that LPS, adalimumab, and cyclosporine in concentration used to determinate expression profile of TGF*β*-related genes affect the vitality of human keratinocyte (*p* > 0.05, one–way ANOVA test). However, when adalimumab in concentration of 80 *μ*g/ml was added to the culture, it was observed that 41.07% cells are viable compared to untreated cells (*p* < 0.05, one–way ANOVA test, post-hoc Tukey test).  Figure 1 shows cytotoxicity assay results.

The second stage of our study was focused on analyzing microarray profile of TGF*β*-related genes.

Out of 22,277 mRNA IDs presented in HG-U133A_2, 2777 mRNA were associated with TGF*β* signaling pathways (Affymetrix NetAffx Analysis Center database after entering the phrase: “tgf beta signaling pathways”, data obtained on 28^th^ February 2020).

The one-way ANOVA test indicated that from 999 mRNA on the significant level *p* < 0.05, 351 mRNA were differentiating keratinocyte culture exposed to LPS in comparison to a control, and for 16 of them, (∣FC | >4.00). In turn, changes in expression of 307 mRNA were statistically significant in cell culture with LPS and adalimumab compared to the control, and 214 mRNA-differentiated HaCaT culture were exposed to LPS and cyclosporine A from the control.

Then, a post-hoc Tukey test was carried out. It allowed indicating the number of mRNA differentiating between the different groups compared. The analysis showed that the number of mRNAs differentiating HACAT cultures exposed to LPS and adalimumab compared with the control for each time of incubation is as follows: H-2 vs C–106 mRNA, H-8 vs C–84 mRNA, and H-24 vs C-11 mRNA. For cultures with LPS and cyclosporine A, the following number of differentiating mRNA may be observed compared to the control: H-2 vs C–111 mRNA, H-8 vs C–57 mRNA, and H-24 vs C-42 mRNA.

The next step was associated with determining the number of mRNA characteristic for 2, 8, and 24 hours exposition to the drug in comparison with the control.

With reference to keratinocytes exposed to LPS and adalimumab, it could be observed that 23 of 106 differentiating mRNA IDs were characteristic for 2-hour long incubation, 12 of 84 after 8 h exposition of the cells to anti-TNF drug, and only 2 of 11 mRNA specifically differentiating after 24 h. It is worth to see that 3 mRNA IDs corresponding to genes: *TGFβ1*, *BMP6*, and *JAK2* were differentiated genes regardless of the incubation time.

In turn, for keratinocyte culture treated with LPS and cyclosporine A, it indicated the following number of differentiating mRNA, 12 mRNA IDs of 111 mRNA IDs after 2 h compared to control and 9 mRNA IDs of 57 mRNA IDs after 8 h exposure of cells to drug, and prolonged incubation time to 24 h showed that 2 mRNA IDs of 42 mRNA IDs were characteristic. In turn, 4 mRNA IDs changed their expression regardless of time: *TGFβ1*, *TGFβRI*, *BMP2*, and *SMAD3*.

Table 3 presents the results of microarray expression profile of genes related to TGF*β* pathways (∣FC | >4.00 from culture exposed to LPS in comparison with the control).

**Figure 2:**
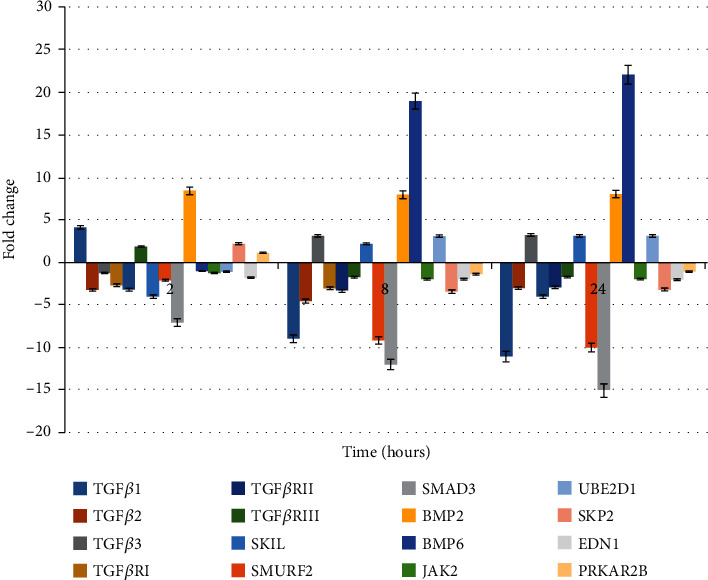
Expression of 14 mRNA in the HaCaT culture treated with LPS+adalimumab (RTqPCR results) presented as fold change in expression when compared to a control culture.

**Figure 3:**
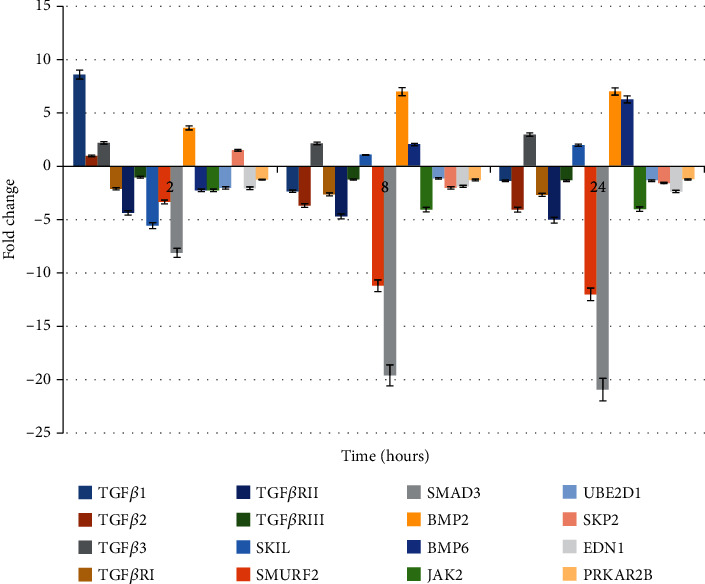
Expression of 14 mRNA in the HaCaT culture treated with LPS+cyclosporine A (RTqPCR results) presented as fold change in expression when compared to a control culture.

In turn, Figures 2 and 3 show the results of transcriptional activity of analysing genes obtained from RTqPCR as a validation of the microarray experiment. The obtained results confirmed differences in microarray expression pattern of evaluating genes.

Next, we assess variances in the level of TGF*β*1, TGF*β*2, JAK2, BMP2, BMP6, SMAD3, and EDN1 on the proteome level in the HaCaT culture treated with LPS and either adalibumab or cyclosporine (Table 4).

It can be observed that the direct changes were similar as observed on the transcriptome level. The statistical analysis indicated that variances in the expression pattern of all analyzed proteins in the HaCaT culture treated with any agent with comparison with the control, untreated cells were statistically significance (*p* < 0.05).

The last step of analysis was carried out using the overrepresentation test with the Bonferroni correction to determine TGF*β*-dependent signaling pathways associated with 16 differentiating keratinocyte genes, in both cultures. The analysis showed that the genes analyzed most closely associated with the TGF beta signaling pathway, oxidative stress response, G2/M transition of mitotic call cycle, SCF-dependent proteasomal ubiquitin-dependent protein catabolic process, JAK/STAT signaling pathway, and interferon-gamma signaling pathway.

## 4. Discussion

There were several reasons for us to study the microarray gene expression profile associated with the signaling pathways induced by TGF*β*. Firstly, in our previous studies, we observed that under the influence of cyclosporin A therapy in patients with psoriasis, there is a change in the transcriptional activity of *TGFβ1-3* and *TGFβRI-III* [17, 18]. Studies have indicated that CsA therapy significantly increases the risk of skin cancer [39, 40]. Therefore, it seems reasonable to better understand how CsA also affects other transcripts of TGF*β*-dependent path genes. Secondly, we also found that adalimumab influences the expression pattern of the *BIRC5* gene [38], encodes survivin, which is not present under physiological conditions. Its expression is found in the case of the cancerous process [41–43]. Like ciclosporin, adalimumab therapy might be associated with an increased risk of skin cancers [44–46], which confirms the need for further research not only on the efficacy but also the safety profile of anti-TNF therapies. Changes in the expression of TGF*β*s and signaling pathways induced by it are observed, e.g., in psoriasis [15–18], in renal fibrosis [47], in bone formation [48], and in the course of tumors [49, 50].

Therefore, in this study, we evaluated not only the effect of cyclosporin A and adalimumab on the expression of TGF*β*-inducted pathways but also compared the effect of both drugs on them. Comparing the microarray expression profile of analysed transcripts in HaCaT culture, it can be concluded that in adalimumab, statistically significant changes in transcriptional activity of a larger number of genes than cyclosporin A were made. Regarding the keratinocytes exposed to adalimumab, this study was focused on genes that, based on the gene ontology, were classified directly on the TGF*β* pathway [51].

*SKIL* encodes the Ski-related novel protein N (SnoN) which is associated with the TGF*β*/SMAD pathway and, as the studies show, also with the pathway dependent on the p53 gene, which plays a key role in carcinogenesis [52, 53]. Cao et al. showed a strong correlation between the expression of SKIL and TGF*β*1 of hepatocellular carcinoma; the level of expression of which was significantly elevated compared to controls [54]. On the other hand, the observations made by Longerich et al. showed no significant role of SKIL in hepatocellular carcinoma [55], which is most probably associated with a smaller number of the study group than in the studies of Cao et al. [54]. In our studies, we found an increase in SKIL expression in keratinocytes exposed to adalimumab compared to controls, which may suggest changes associated with neoplastic transformation. The effect of silencing the SKIL gene expression can also be caused directly, activated by TGF*β* [56] and SMAD3 [57–60], overexpressed in our study. However, with respect to the *SMURF2* gene belonging to the HECT family of E3 ubiquitin ligases, Smad ubiquitination regulators, the negative regulator of the TGF*β* pathway [61–63], we observed a decrease in its level after administration of the anti-TNF drug, confirming the therapeutic effect of adalimumab. Zhao et al. demonstrated that TNF-*α* overexpresses SMURF2 while reducing transcripts encoding E-cadherin promoting epithelial-mesenchymal transition (EMT). [64]. It has also been confirmed that biological treatment restores the balance between SMURF2 and TNF-*α* expression [65]. Thus, considering the simultaneous expression of these three genes and the time from the introduction of the drug into the culture, it can be concluded that the observed changes may also result from the activation of adaptation mechanisms associated with the changing TNF-*α* concentration in the presence of adalimumab and the aspiration of the homeostatic system. In turn, after 8 hours of HACAT incubation with the drug, we found overexpression of *BMP2* and *BMP6* (bone morphogenetic protein 2, 6) with simultaneous silencing of TGF*β*RIII expression; GDF5 (cartilage-derived morphogenetic protein 1) was involved in the differentiation of joint forming cells and interstitial spaces [66]. Chen et al. and Joo et al. emphasise, respectively, that the increase in the expression of BMP2 and BMP6 positively correlates with the severity of changes observed in ankylosing spondylitis [67, 68]. In addition, studies by Chen et al. indicated that the change in the expression profile of BMP2 resulted directly from the exposure of PBMCs to TNF-*α* [67]. Therefore, it seems that the observation of nearly 7- and 3-fold increase in the BMP2 and BMP6 transcriptional activities suggests that adalimumab did not adequately neutralize TNF-*α*. One should also not forget that cytokines are characterized by pleiotropic activity [69]. However, the study by Eguchi et al. should be taken into account, who also noted the overexpression of BMP2 under the influence of anti-TNF therapy (etanercept), which considered the beneficial effect of therapy [70]. The decrease in the *GDF5* (growth differentiation factor-5) transcriptional activity observed by us is convergent with the observations of Liu et al. who observed the silencing of its expression in diseases occurring with peripheral joint degradation. They emphasize that the observed expression profile of GDF5 is the result of the action of TNF-*α* and IL-1*β*, which inhibit the secretion of GDF5 [71, 72]. This is further evidence of the gradual disappearance of adalimumab and loss of keratinocytes susceptibility to anti-TNF. Therefore, regular, continuous intake of a TNF-*α* inhibitor is appropriate to maintain adequately low TNF-*α* levels. Comparing the level of *TGFβRIII* in HACAT culture with adalimumab and in patients treated with cyclosporine A [17], the same direction of change and decrease in activity during the course of treatment can be seen.

JAK2 (Janus kinase 2) silencing during molecular-targeted therapy, including adalimumab has been observed in both Taylor et al. [73] and Vollenhoven et al. [74]. It should also be emphasized that the JAK2 expression profile indicates that adalimumab counteracts EMT [75], which is consistent with the observations in HaCaT culture exposed to the anti-TNF drug. UBE2D1 is associated with the TGF*β*/ubiquitin proteasome pathway [74]. Koné-Pau et al. indicated that ubiquitination and degradation in TNFR1 proteasomes is one of the key factors of inhibition of the NF*κ*B pathway, contributing to the reduction of inflammation [76]. Kawamoto et al. indicated that ubiquitination is a process controlled by NOTCH and TNF-*α* pathways and that infliximab (TNF-*α* inhibitor) therapy in the course of inflammatory bowel diseases results in a reduction of UBD at the protein level [77]. The *UBE2D1* expression profile that is different from our profile may result from the use of other anti-TNF drugs and their different construction and other research model.

Our analysis indicated that among the genes differentiating the culture exposed to cyclosporin A from control, the *SKP2* (S phase kinase-associated protein), *EDN1* (endothelin 1), and *PRKAR2B* (protein kinase cAMP-dependent type II regulatory subunit beta) genes showed association based on the analysis of the PANTHER 14.1 program. For all of these transcripts, a decrease in activity was observed compared to untreated cells.

Changes in the transcriptional activity of the *SKP2* gene were analyzed most often in the context of carcinogenesis. It is indicated that this gene may become a new molecular marker of the carcinogenesis process, because its overexpression is associated with a worse prognosis [78–80]. Also in relation to *PRKAR2B*, we found that CsA reduced the level of its expression, which is consistent with the observations of Zgheib et al. [81]. This gene encodes regulatory subunit of the cAMP-dependent protein kinases [82]; the increased expression of which is associated with the tumor cells obtaining a higher metastatic potential by induction of EMT [83, 84]. Therefore, the silencing of *SKP2* and *PRKR2B* after the addition of cyclosporin A may confirm its anti-inflammatory properties and efficacy. In addition, it can be assumed that in the described relationship between CsA therapy and an increased risk of cancer [39, 40], the *SKP2* and *PRKR2B* genes are not involved or their role is marginal.

When assessing the pattern of expression of *EDN1* under the influence of CsA, we found that it is different from that observed in HaCaT culture exposed to adalimumab [85]. Considering the observation by Chen et al. that hypoxvia induces overexpression of *EDN1* [86] for 8 hours and that methotrexate inhibits endothelin activity [87], silencing the expression of *EDN1* confirms the anti-inflammatory properties of CsA and indirectly indicates the safety of its use [88]; the phenomenon of hypoxia is associated with the induction of neoplastic transformation [89, 90].

In summary, the regularity of taking the drug is important for the efficacy of psoriasis therapy. It should also take into account the regular observation of patients, monitoring the effectiveness of therapy and the possibility of adverse events during therapy with adalimumab and cyclosporin A.

## 5. Conclusions

Analysis of the expression profile of genes associated with TGF*β* signaling pathways has demonstrated the potential of cyclosporin A and adalimumab to induce changes in their transcriptional activity. The anti-TNF drug seems to affect TGF*β* cascades to a greater extent than cyclosporin A. The evaluation of the microarray profile indicated the efficacy and safety of both drugs being used in keratinocyte cell culture.

## Figures and Tables

**Table 1:** The nucleotide sequence of primers used to amplify selected genes by RTqPCR reaction.

mRNA	Primers sequence
*TGFβ1*	Forward: 5′TGAACCGGCCTTTCCTGCTTCTCATG3 ′
	Reverse: 5′GCGGAAGTCAATGTACAGCTGCCGC3 ′
*TGFβ2*	Forward: 5′TACTACGCCAAGGAGGTTTACAAA3 ′
	Reverse: 5′TTGTTCAGGCACTCTGGCTTT3 ′
*TGFβ3*	Forward: 5′CTGGATTGTGGTTCCATGCA3 ′
	Reverse: 5′TCCCCGAATGCCTCACAT3 ′
*TGFβR1*	Forward: 5 ′-ACTGGCAGCTGTCATTGCTGGACCAG-3 ′
	Reverse: 5 ′-CTGAGCCAGAACCTGACGTTGTCATATCA-3 ′
*TGFβR2*	Forward: 5 ′-GGCTCAACCACCAGGGCATCCAGAT-3 ′
	Reverse: 5 ′-CTCCCCGAGAGCCTGTCCAGATGCT-3 ′
*TGFβR3*	Forward: 5′-ACCGTGATGGGCATTGCGTTTGCA-3′
	Reverse: 5 ′-GTGCTCTGCGTGCTGCCGATGCTGT-3 ′
*SKIL*	Forward: 5′-AGAGACTCTGTTTGCCCCAA-3′
	Reverse: 5′-CAGGATGGGGCATTGAATGG-3′
*SMURF2*	Forward: 5 ′-CGGCAAGAACTTTCCCAACA-3 ′
	Reverse: 5 ′-GCAACGCCTCCATAGTCAAG-3 ′
*SMAD3*	Forward: 5 ′-CTCTGGGTGCTTGGGAACTA-3 ′
	Reverse: 5 ′-ATCCAAATGCAGCCAAACGT-3 ′
*BMP2*	Forward: 5 ′-AATGCAAGCAGGTGGGAAAG-3 ′
	Reverse: 5 ′-GCTGTGTTCATCTTGGTGCA-3 ′
*BMP6*	Forward: 5 ′-AGAAGAAGGCTGGCTGGAAT-3 ′
	Reverse: 5 ′-GAAGGGCTGCTTGTCGTAAG-3 ′
*JAK2*	Forward: 5 ′-AGTAAAAGTCCACCAGCGGA-3 ′
	Reverse: 5 ′-AGGAGGGGCGTTGATTTACA-3 ′
*UBE2D1*	Forward: 5 ′-ATCCACCTGCTCACTGTTCA-3 ′
	Reverse: 5 ′-GTGACCATTGTGACCTCAGA-3 ′
*SKP2*	Forward: 5 ′-TTCACGTCATTCTCCTGCCT-3 ′
	Reverse: 5 ′-CATGCCTGTAATCCCAGCAC-3 ′
*EDN1*	Forward: 5 ′-ATCCTCTGCTGGTTCCTGAC-3 ′
	Reverse: 5 ′-AGGTCCATTGTCATCCCCAG-3 ′
*PRKAR2B*	Forward: 5 ′-CTCAGGGAGATTCGGCTGAT-3 ′
	Reverse: 5′-ATTTGACAGTCCCAATGGCG-3 ′
*ACTB*	Forward: 5 ′-GAAGGTGAAGGTCGGAGTC-3 ′
	Reverse: 5 ′-GAAGATGGTGATGGGATTC-3 ′

**Table 2:** Names of kit reagents used to determinate the level of selected protein by ELISA assay.

Protein	Name of the kit	Catalog number
TGF*β*1	TGF beta-1 Human ELISA Kit	BMS249-4
TGF*β*2	TGF beta-2 Human ELISA Kit	BMS254
JAK2	JAK2 (total) Human ELISA Kit	KHO5521
BMP2	BMP-2 Human ELISA Kit	EHBMP2
BMP6	BMP-6 Human ELISA Kit	EHBMP6
EDN1	Endothelin-1 (ET-1) Human ELISA Kit	EIAET1

**Table 3:** Microarray profile of TGF*β*-dependent genes in HaCaT culture exposed to LPS or LPS+adalimumab/cyclosporine A (∣FC | >4.00 in the first comparison).

Group		LPS	LPS+adalimumab			LPS+cyclosporine A		
		H-8 vs C	H-2 vs C	H-8 vs C	H-24 vs C	H-2 vs C	H-8 vs C	H-24 vs C
ID	MRna							
203085_s_at	*TGFβ1*	12.11	4.16	-8.36	-11.07	8.66	-2.33	-1.33
220407_s_at	*TGFβ2*	4.14	-3.28	-4.45	-3.02	1.01	-3.66	-4.06
209747_at	*TGFβ3*	-4.99	-1.22	3.16	3.22	2.22	2.18	3.01
206943_at	*TGFβRI*	4.03	-2.99	-3.05	-4.01	-2.1	-2.62	-2.66
207334_s_at	*TGFβRII*	4.15	-3.21	-3.33	-2.96	-4.36	-4.69	-5.02
204731_at	*TGFβRIII*	4.18	1.85	-1.74	-1.72	-1.11	-1.22	-1.31
206675_s_at	*SKIL*	-7.26	-4.02	2.23	3.11	-5.22	1.11	2.01
205596_s_a	*SMURF2*	7.09	-2.11	-9.14	-9.19	-3.39	-10.21	12.01
205397_x_at	*SMAD3*	18.96	-7.01	-11.99	-15.03	-8.13	19.11	-20.94
205289_at	*BMP2*	-11.55	8.01	7.96	8.12	3.62	7.05	7.06
206176_at	*BMP6*	-14.02	+1.01	19.06	22.05	-2.24	2.09	6.33
205842_s_at	*JAK2*	4.36	-1.25	-1.98	-2.06	-2.22	-4.08	-4.01
214590_s_at	*UBE2D1*	-3.88	-1.11	3.08	3.14	-2	1.11	-1.33
210567_s_at	*SKP2*	5.02	2.62	-3.42	-3.22	1.79	-2.33	-1.17
218995_s_at	*EDN1*	4.14	-1.48	-1.95	-2.07	-2.03	-1.99	-2.74
203680_at	*PRKAR2B*	4.29	1.22	-1.5	-1.11	-1.22	-1.32	-1.18

+ indicates overexpression of gene (increased level of mRNAs); - indicates suppressed gene expression (decreased level of mRNAs); ID: ID of the probe on a microarray; FC: fold change; C: control culture; H-2, H-8, and H-24: time of exposure to the medicine.

**Table 4:** Changes in the level of selected proteins in cell culture exposed to LPS, LPS+adalimumab, LP+cyclosporine A, and a control culture.

Protein	Control	LPS	LPS+adalimumab			LPS+cyclosporine A		
	H-0	H-8	H-2	H-8	H-24	H-2	H-8	H-24
TGF*β*1 (pg/ml)	396.85	1159.33	741.04	455.01	211.06	799.14	521.11	341.06
TGF*β*2 (pg/ml)	32.85	211.09	110.65	77.01	36.84	144.09	100.27	42.11
JAK2 (ng/ml)	2.22	27.11	12.44	8.01	3.12	11.02	6.37	3.18
BMP2 (pg/ml)	52.09	74.04	369.88	2104.44	2096.58	758.95	1985.09	2145.01
BMP6 (pg/ml)	36411.43	411.08	8856.02	10651.55	13751.06	2214.08	4648.12	10258.09
EDN1 (pg/ml)	12.67	33.25	22.47	21.01	18.25	19.24	18.78	18.96

## Data Availability

The data used to support the findings of this study are included in the article. The data will not be shared due to the third-party rights and commercial confidentiality.
